# Biodegradation of Di-*n*-Butyl Phthalate by a Newly Isolated Halotolerant *Sphingobium* sp.

**DOI:** 10.3390/ijms141224046

**Published:** 2013-12-10

**Authors:** Decai Jin, Xiao Kong, Bingjian Cui, Zhihui Bai, Hongxun Zhang

**Affiliations:** 1 College of Resources and Environment, University of Chinese Academy of Sciences, Beijing 100049, China; E-Mails: jindecai19841019@gmail.com (D.J.); cuibingjian12b@mails.ucas.ac.cn (B.C.); hxzhang@ucas.ac.cn (H.Z.); 2 Research Centre for Eco-Environmental Sciences, Chinese Academy of Sciences, Beijing 100085, China; E-Mail: kongxiaozaikeda@163.com; 3 Qingdao University of Science and Technology, Qingdao 266042, China

**Keywords:** di-*n*-butyl phthalate, biodegradation, halotolerant, *Sphingobium* sp., 16S rRNA gene, *gyrb* gene

## Abstract

A Gram-negative strain (TJ) capable of growing aerobically on mixed phthalate esters (PAEs) as the sole carbon and energy source was isolated from the Haihe estuary, Tianjin, China. It was identified as belonging to the *Sphingobium* genus on the basis of morphological and physiological characteristics and 16S rRNA and *gyrb* gene sequencing. The batch tests for biodegradation of di-*n*-butyl phthalate (DBP) by the *Sphingobium* sp. TJ showed that the optimum conditions were 30 °C, pH 7.0, and the absence of NaCl. Stain TJ could tolerate up to 4% NaCl in minimal salt medium supplemented with DBP, although the DBP degradation rates slowed as NaCl concentration increased. In addition, substrate tests showed that strain TJ could utilize shorter side-chained PAEs, such as dimethyl phthalate and diethyl phthalate, but could not metabolize long-chained PAEs, such as di-*n*-octyl phthalate, diisooctyl phthalate, and di-(2-ethyl-hexyl) phthalate. To our knowledge, this is the first report on the biodegradation characteristics of DBP by a member of the *Sphingobium* genus.

## 1. Introduction

Phthalate esters (PAEs) are a class of refractory organic compounds that are widely used in plastics, coatings, and cosmetics [1]. Because PAEs are not chemically bound to the products to which they are added, they can easily migrate into the environment during production, use, and disposal [2]. PAEs have now been detected in almost every environment, including water, air, industrial effluents, soil, and food [3]. PAEs are recalcitrant compounds that have accumulated as environmental contaminants. In recent years, some PAEs have received increasing attention because they are estrogenic endocrine-disrupting compounds that can have toxic effects on reproductive development, even at very low concentrations [4]. The United States Environmental Protection Agency (US EPA 1992) and China National Environmental Monitoring Centre have classified most of the PAEs, such as diethyl phthalate (DEP), benzyl butyl phthalate (BBP), DBP, and di-(2-ethyl-hexyl) phthalate (DEHP) as priority pollutants [5].

The degradation of phthalates in the environment can occur by hydrolysis, photolysis, and biodegradation. Numerous studies have demonstrated that metabolic breakdown of PAEs by microorganisms plays a major role in the environmental degradation of these widespread pollutants due to low rates of chemical hydrolysis and photolysis in the environment [6]. Screening of microorganisms for phthalate biodegradation potential is a critical step in constructing an effective remediation system for minimizing their adverse effects.

Many microorganisms with the ability to utilize PAEs and their isomers as their sole carbon and energy source have been isolated from environments such as activated sludge, mangrove sediment, and wastewater; these include *Agrobacterium* sp. [7], *Rhodococcus* sp. [8], *Deinococcus radiodurans*, *Pseudomonas stutzeri* [9], and Cyanobacteria [10]. However, most of the microorganisms have been isolated from terrestrial subsurface environments, and far less is known about their counterparts growing in the saline environments. Investigating what types of marine bacteria can remove PAEs in a salty environment could prove to be a valuable area of research.

In the present study, we characterized a potent new DBP-degrading bacterial strain isolated from the Haihe estuary. To determine the optimal conditions for degradation, we investigated the effects of temperature, pH, and salt concentration on the DBP biodegradation performance of strain TJ. Such evaluations of degradation capacity and substrate utilization could potentially serve as a guide to assist DBP remediation efforts in such environments.

## 2. Results and Discussion

### 2.1. Identification and Characterization of the PAEs Degrading Strain

**Figure 1.**
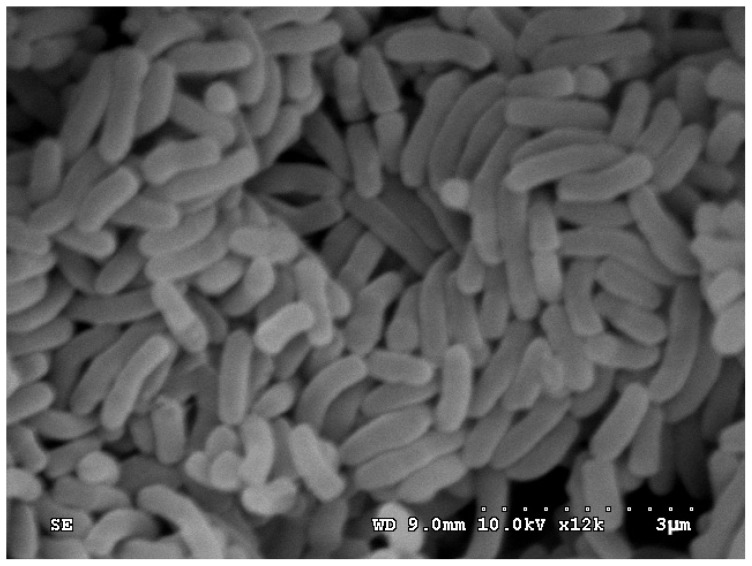
Scanning electron micrograph of *Sphingobium* sp. TJ (×10,000).

Viable bacteria were isolated from water samples collected from the Haihe estuary after acclimation to PAEs for approximately 2 months in an aerated basin. The screening tests isolated one pure bacterial strain as the dominant PAE-degrading strain, which we named as strain TJ. The strain had a short-rod shape, a size of (0.8–1.5) μm × (0.3–0.5) μm, was Gram negative, and non-flagellated. The morphological features of strain TJ are shown in Figure 1.

**Figure 2.**
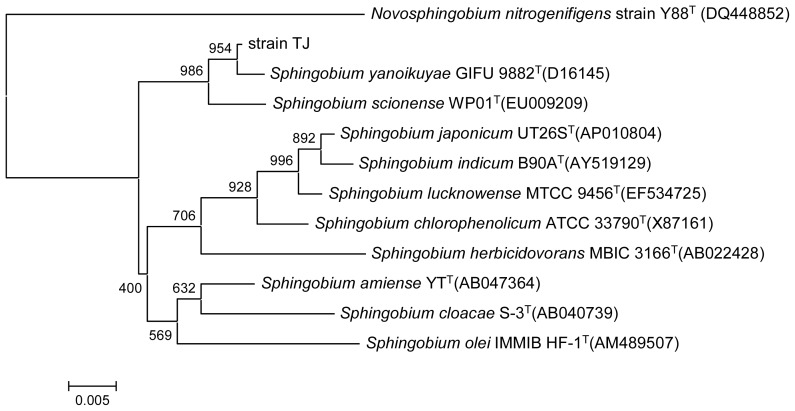
Phylogenetic tree derived from the 16S rRNA gene sequence of strain TJ and sequences from related species. Distances were calculated using the neighbor-joining method. Numbers at branch points are bootstrap values based on 1000 samplings. *Novosphingobium nitrogenifigens* strain Y88^T^ (DQ448852) was used as the outgroup.

The colonies of the strain cultured on Luria-Bertani agar for 24 h were light yellow and round. The strain tested positive for catalase but was negative for oxidase activity, nitrate reduction, gelatin hydrolysis, and methyl red and Voges Proskauer’s reactions. It was able to utilize glucose, lactose, galactose, fructose, maltose, sucrose, mannose, histidine, valine, leucine, and tyrosine as sole carbon and energy sources. Manioc, glycine, serine, threonine, and methionine were not utilized. Comparison of 16S rRNA gene sequences showed that strain TJ (GenBank accession number KF672731) had 99.6% similarity with *Sphingobium yanoikuyae* GIFU 9882^T^ (Figure 2). This result was confirmed by sequence analysis of the *gyrb* gene (GenBank accession number KF672732), which showed the highest sequence similarity (87.8%) with *Sphingobium chlorophenolicum* (GenBank accession number CP002798). Taken together, the morphological, physiological, biochemical, and genetic sequence analyses identified strain TJ as belonging to the genus *Sphingobium*.

### 2.2. Substrate Utilization Tests

The substrate utilization tests indicated that the isolate had differing abilities for degrading different phthalate esters (Table 1). The strain grew well in media containing dimethyl phthalate (DMP), DEP, or DBP, but could not utilize longer alkyl-chained PAEs such as di-*n*-octyl phthalate (DOP) and diisooctyl phthalate (DIOP). This result demonstrated that the side chain of the substrates has a significant effect on the ability of strain TJ to biodegrade PAEs, which is consistent with the results obtained with *Ochrobactrum* sp. JDC-41 [11]. Furthermore, although strain TJ could utilize mono-butyl phthalate (MBP) and benzoic acid, it was unable to utilize two other common intermediate products, phthalic acid and protocatechuic acid. Based on these results, DBP, one of most popular plasticizers, was selected for further degradation tests.

Although the degradation of PAEs by microorganisms from different environments has been described, no studies have been performed on DBP degradation by pure cultures of *Sphingobium* spp. However, many species in the *Sphingobium* genus have been reported to degrade a variety of other pollutants such as lindane [12], isoproturon [13], and pentachlorophenol [14]. These reports indicated that the *Sphingobium* genus may play an important role in the bioremediation of environmental pollutants.

### 2.3. Effects of pH and Temperature on Microbial Growth and DBP Degradation

**Figure 3.**
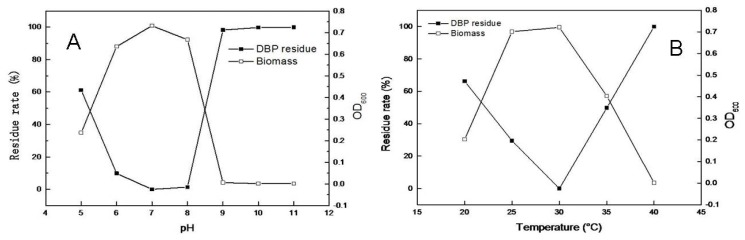
Effects of pH and temperature on strain TJ biomass and DBP biodegradation. (**A**) Temperature and biomass after 32 h growth at 30 °C for various pH values; and (**B**) Temperature and biomass after 32 h growth at pH 7.0 at various temperatures.

The time-courses of microbial growth and DBP degradation with an initial concentration of 500 mg/L were determined at 150 rpm at 30 °C in the dark. As shown in Figure 3A, all of the DBP was degraded after incubation for 32 h at pH 7.0, and an increase in biomass corresponding to the decrease of DBP was observed. The strain TJ was sensitive to pH levels above 9.0, degrading almost no DBP when grown at pH 9.0, 10.0 or 11.0.

Many previous studies have shown that temperature is also an important factor influencing PAE degradation. As shown in Figure 3B, the DBP degradation rate increased rapidly as the temperature increased from 20 to 30 °C. The highest DBP degradation rate (100%) and maximum biomass for strain TJ were achieved at 30 °C. The rate of degradation by strain TJ dropped significantly when the temperature was increased from 30 to 40 °C, with almost no degradation occurring at 40 °C.

### 2.4. Effects of NaCl Induction on Microbial Growth and DBP Degradation

**Figure 4.**
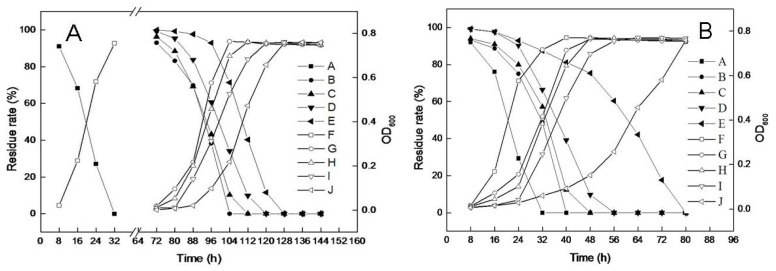
Effect of NaCl concentration on biomass and DBP biodegradation by strain TJ. (**A**) Without NaCl induction in minimal salt medium (MSM) contains DBP; (**B**) With 3% NaCl induction in MSM contains DBP. A–E represent DBP residue levels at NaCl concentrations of 0%, 1%, 2%, 3%, and 4%, respectively; F–J represent strain TJ biomass at NaCl concentrations of 0%, 1%, 2%, 3%, and 4%, respectively.

Salinity is a significant parameter in the ocean ecosystem. Because strain TJ was isolated from a marine environment, the effect of salt concentration on DBP biodegradation was also tested. As shown in Figure 4A, strain TJ was sensitive to the salinity level. No DBP residue remained after incubation for 32 h when NaCl was not present, reflecting the complete degradation of DBP. The degradation rate slowed when salt concentrations were increased to 1%–4%, and virtually no DBP degradation or bacterial growth was observed before 72 h. The lag phase for DBP degradation becomes longer as NaCl concentration increased. As shown in Figure 4B, after induction by NaCl, the ability of strain TJ to degrade DBP in the presence of NaCl improved significantly; all of the DBP was degraded within 80 h, even at the 4% NaCl concentration.

Recently, many bacterial strains have been isolated from various environments, but only a little research has focused on PAE degradation in the presence of salt. Wang *et al.* (2008) isolated *Burkholderia cepacia* DA2 from marine sediment in the South China Sea; this species could degrade DMP when grown in the salinity range of 0%–1%, and its lag phase also increased with increase in salinity [15]. Although strain TJ exhibited a similar trend in that higher salinity levels reduced the bacterial growth rates, resulting in longer degradation times, it could tolerate a NaCl concentration up to 4%. This characteristic suggests that strain TJ would be a promising candidate for the bioremediation of DBP-contaminated wastewater containing salts.3. Experimental Section

### 3.1. Chemicals

DMP, DEP, DBP, DOP, and DIOP were purchased from Alfa Aesar (Ward Hill, MA, USA); all were >98% pure. High-performance liquid chromatography-grade methanol was purchased from Sigma (Cream Ridge, NJ, USA). All other chemicals and solvents were of analytical-grade quality.

### 3.2. Enrichment Culture and Isolation of Bacteria

A water sample with a salinity of approximately 3% was collected from the Haihe estuary located at north latitude 38.98°, east longitude 117.73°. A 1 mL water sample was mixed with 100 mL of a minimal salt medium (5.8 g/L K_2_HPO_4_, 4.5 g/L KH_2_PO_4_, 2.0 g/L (NH_4_)_2_SO_4_, 0.16 g/L MgCl_2_, 0.02 g/L CaCl_2_, 0.0024 g/L Na_2_MoO_4_·2H_2_O, 0.0018 g/L FeCl_3_ and 0.0015 g/L MnCl_2_·2H_2_O in double-distilled water) in an aerated basin at room temperature, and DMP, DEP, DBP, DOP, and DIOP were added in equal proportions to act as the sole carbon and energy source. The total concentration of PAEs was increased gradually from 50 to 500 mg/L. After acclimation for 10 weeks, the enriched medium was used to inoculate nutrient agar plates under aseptic conditions. The plates were incubated at 30 °C, and colonies were observed after 48 h. Pure clones were obtained by the streak-plate technique.

### 3.3. Identification of a Pure Culture

Conventional physiological and biochemical characterizations for determining the tetrahydrofuran-degrading bacterium were performed as described by Yoon and Oh [16]. Chromosomal DNA from the isolated bacteria was extracted using an EZ-10 spin column genomic DNA miniprep kit (Bio Basic Inc., Markham, ON, Canada) according to the manufacturer instructions. The *gyrb* and 16S rRNA genes were amplified using the bacterial primers UP1 and UP2 [17] and F27 and R1492 [18], respectively. The PCR products were cloned into the pGEM-T Easy vector (Promega, Madison, WI, USA) and sequenced. Phylogenetic and distance analyses of the aligned sequences were performed using the MEGA software (version 4.1; The Biodesign Institute, Tempe, AZ, USA). The resulting unrooted tree topologies were constructed using the neighbor-joining method with bootstrapping.

### 3.4. Substrate Utilization Tests

For substrate utilization tests, the isolated strain was inoculated into MSM containing 200 mg/L of one of the following substrates: DMP, DEP, DBP, DOP, DIOP, MBP, phthalic acid, protocatechuic acid, or benzoic acid. Uninoculated media with each substrate were used as negative controls. Substrate utilization was based on microbial growth as indicated by an increase in biomass ascertained by OD_600_ measurements.

### 3.5. Biodegradation of DBP

The isolated strain was grown in MSM containing DBP (500 mg/L) for 28 h at 30 °C on a rotary shaker (150 rpm). NaCl-induced cells were grown in the same medium supplemented with 3% NaCl and were incubated for 100 h. Next, the cells were harvested and washed three times with 20 mM phosphate buffer (pH 7.0) and resuspended in the same buffer at an OD_600_ of 0.2. One millilitre of the prepared cell suspension was then inoculated into the medium for the biodegradation test. The degradation experiments were performed in 50 mL Erlenmeyer flasks with an initial concentration of 500 mg/L for DBP. The extractions and quantifications were performed as described by Jin *et al.* [19]. All experiments were performed in triplicate.

## 4. Conclusions

In summary, a bacterial strain (TJ) capable of utilizing DBP as its sole source of carbon and energy was isolated from water samples collected from the Haihe estuary. Strain TJ was identified as belonging to the *Sphingobium* genus based on the analyses of morphological features, physiochemical properties, and 16S rRNA and *gyrb* gene sequences. To our knowledge, this is the first report of DBP degradation by a member of the *Sphingobium* genus. The strain can also tolerate NaCl concentrations up to 4%. These results may have direct relevance for the bioremediation of DBP pollutants in contaminated wastewater containing salts.

## Figures and Tables

**Table 1.** Substrate utilization profile for strain TJ.

Substrate	Utilization	Substrate	Utilization	Substrate	Utilization
DMP	+	DOP	−	Phthalic acid	−
DEP	+	DIOP	−	Protocatechuic acid	−
DBP	+	MBP	+	Benzoic acid	+

“+” denotes positive; “−” denotes negative; DMP = dimethyl phthalate; DEP = diethyl phthalate; DBP = di-*n*-butyl phthalate.
